# Challenges and recommendations for advancing respite care for families of children and youth with special health care needs: A qualitative exploration

**DOI:** 10.1111/hex.13831

**Published:** 2023-09-13

**Authors:** Roberta L. Woodgate, Corinne A. Isaak, Ardelle Kipling, Sue Kirk

**Affiliations:** ^1^ College of Nursing Rady Faculty of Health Sciences, University of Manitoba Winnipeg Canada; ^2^ School of Nursing University of Manchester Manchester UK

**Keywords:** children, CYSHCN, process maps, qualitative, respite, service providers, special health care needs, youth

## Abstract

**Introduction:**

Caring for children and youth with special health care needs (CYSHCN) is a significant undertaking for families. While respite care is intended to address this burden, demand continues to exceed supply. Exploring the perspectives of respite service providers (SPs) and stakeholders (SKs) provides unique insight into families' needs and respite care systems.

**Methods:**

We conducted semistructured interviews with 41 respite care SPs and SKs across four Canadian provinces to ascertain perspectives on current and ideal respite care for families of CYSHCN. The analysis included delineating units of meaning from the data, clustering units of meaning to form thematic statements and extracting themes. The second‐level analysis involved applying themes and subthemes to cross‐functional process maps.

**Findings:**

Participants noted the critical, but sometimes absent role of Community Service Workers, who have the ability to support families accessing and navigating respite care systems. SPs and SKs identified current respite systems as operating in crisis mode. New findings suggest an ideal respite care system would incorporate advocacy *for* families, empower families and value CYSHCN, their families and respite workers.

**Conclusion:**

The evidence of unmet respite care needs of families of CYSHCN across Canada has long been available. Our findings identifying respite system challenges and solutions can be used by funders and policymakers for planning and enhancing resources, and by healthcare professionals, respite care providers and SKs to understand barriers and take action to improve respite outcomes to meet the respite needs of all families and CYSHCN.

**Patient or Public Contribution:**

The research team is composed of patients, researchers, clinicians and decision‐makers along with our Family Advisory Committee (FAC) composed of members of families of CYSHNC. The FAC was formed and met regularly with research team members, knowledge users and collaborators throughout the study to provide input on design, review themes and ensure findings are translated and disseminated in a meaningful way.

## 1 INTRODUCTION

The number of children and youth with special health care needs^1^ (CYSHCN) in Canada is increasing, due to medical and technological advances,^2^ thus increasing demand for extensive medical therapies, and formal and informal services.^2^, ^3^ While there is no agreed‐upon definition, here, we use the term CYSHCN which includes infants, children and youth with one or more chronic physical, developmental, behavioural or emotional conditions, and require special health and support services.

Caring for CYSHCN is an enormous responsibility for families, which is the premise of respite care services, intended to provide families with short breaks from ongoing caregiving duties, and be beneficial for caregivers, siblings and CYSHCN themselves.^4^, ^5^ Indeed, the *Convention on the Rights of Persons with Disabilities* ^6^ and *United Nations Convention on the Rights of the Child* ^7^ stipulate the rights of children with disabilities to receive adequate care and supports to maintain family life.^8^

Respite care services vary across Canada both inter‐ and intraprovincially. Although respite may be for crisis intervention, ideally it is part of a supportive network for caregivers and CYSHCN.^9^ In Manitoba (MB), Canada, for example, the provincially administered Children's disABILITY Services programme funds respite for families with CYSHCN under age 18 who have specified disability diagnoses. Depending on eligibility, assessed needs and available resources, respite may be provided via self‐administered, agency‐delivered or direct service providers (SPs).^8^, ^10^

However, research relating to Canadian families with CYSHCN primarily reports moderate to low receipt of respite care. For instance, in British Columbia, 53% of families of CYSHCN had access to publicly funded respite care.^11^ In one Alberta study, 2.5% of the full sample and 26% of CYSHCN with high severity of disability used family disability services (included respite),^12^ whereas, in another survey with 57 families in a large Western Canadian city, 73% used in‐home formal respite care; but 77% of all respondents perceived they were not getting adequate breaks, regardless of the level of care for their CYSHCN.^13^ In a MB study using administrative data, 23.7% of CYSHCN had received respite during the 5‐year study period.^14^ Further, of the 2049 families approved for publicly funded respite in MB in 2019–2020, only 30.8% were able to receive respite during this time period, due for example to being unable to find respite workers, or unaware of alternatives to meet needs.^8^ In Ontario studies, 46% of caregivers of children with cerebral palsy,^15^ and 49.5% of families with CYSHCN had received respite care services in the past 12 months.^16^

Yet for many families with CYSHCN, demand for suitable respite care continues to exceed supply, resulting in unmet respite care needs,^17^, ^18^ and issues with accessibility, availability and affordability^9^, ^19^, ^20^, ^21^, ^22^ including for Canadian families with CYSHCN.^23^, ^24^, ^25^ Furthermore, lack of appropriate skills and experience amongst respite workers, and inconsistent and unregulated training for respite workers can leave parents feeling even more burdened^26^ and impact care provision.^27^

Given families' concerns, it is important to explore the perspectives of respite SPs and stakeholders (SKs) regarding respite care. Previous research reported that respite SP noted challenges with system accessibility and navigation,^22^ funding inflexibilities,^28^ service provision limitations and inefficiencies,^29^ including inadequate supply of,^30^ and training for respite workers,^26^, ^28^, ^29^, ^31^ and regional inequities in service provision.^29^, ^32^ The lengthy list of respite care system challenges is concerning given unmet respite care needs,^33^ which may lead to hospitalization of CYSHCN.^17^ To address these challenges, respite SPs have identified the need for; ‘a one‐stop clinic for families’^29^ ^,p.2554^ effective communication between SPs/teams and families,^22^, ^26^, ^29^ clearly defined respite worker roles,^34^ together with calls for culturally responsive service processes^22^ and person‐ and family‐centred approaches.^26^

The goal of this study was to gather evidence to inform respite care that is responsive and integrative for families of CYSHCN in MB, a Canadian province. This paper presents findings from interviews with respite SPs and SKs, highlighting current challenges within respite care systems along with recommendations to inform an ideal system of respite care using process maps.^35^, ^36^

## 2 METHODS

### 2.1 Study design and participant selection

This qualitative study is part of a mixed methods project, including interviews with MB families (mothers, fathers and siblings) of CYSHCN,^37^ and a population‐based component to assess respite services received/utilized by MB families of CYSHCN.^14^

The interview guide explored participant perspectives on delivering respite care to families. Attention was given to their views on conditions that promote respite care that is responsive and integrative. Similar questions were asked as those asked during the family interviews^37^ but tailored to fit with the nature of the SPs' and SKs' roles (see Supporting Information: Appendix A).

Using a combination of purposive and snowball sampling, we recruited respite SPs (e.g., respite workers, clinician, coordinator) from seven government, health and community organizations across MB, and SKs (e.g., policymakers, administrators, family and advocacy group members) from nine respite services departments/organizations in Canadian provinces using posters, invitation letters, and social media until theoretical saturation was achieved.^38^, ^39^ SKs outside of MB were included to provide a broader scope of recommendations. Interested participants were contacted by study personnel to explain the study and arrange for an interview.

Process mapping was used as a methodological tool to identify current respite system complexities and bottlenecks, and opportunities for improvement.^40^ Process maps portray the multiple roles and steps involved in processes,^40^, ^41^ assisting in ‘…fostering shared understanding of current state and ideal future state processes’.^42^

### 2.2 Data collection

Demographic forms were completed by participants followed by interviews in English, conducted between September 2019 and January 2022 by trained research personnel either in‐person, by phone or video conferencing using various strategies to facilitate discussion.^43^, ^44^ Interviews were audio‐recorded and transcribed verbatim and field notes were recorded to describe nonverbal behaviours of participants, interview dynamics and context.

### 2.3 Analysis

Data analysis occurred simultaneously with data collection. The analysis first involved delineating units of meaning from the data, clustering units of meaning to form thematic statements and extracting themes,^39^, ^45^, ^46^ which were organized into a table of contents in Microsoft Word. All data were reviewed repeatedly for significant statements by authors R. L. W., C. A. I. and A. K. in an attempt to understand participants' lived experiences and meanings through themes.^45^ Any discrepancies or uncertainty of themes were resolved via discussion among all three authors until consensus was achieved. The Family Advisory Committee was presented with findings and reviewed the major themes on an ongoing basis. After meetings, notes were made and assumptions checked to ensure there was no researcher bias. Participants' home/work province are not identified in the quotes to protect confidentiality. Demographic characteristics were calculated using basic descriptive statistics using *SAS® version 9.4*.^47^ In a second level of analysis, themes and subthemes were applied to cross‐functional process maps,^48^ depicting current state and ideal state respite care systems.

## 3 RESULTS

### 3.1 Participants

Participant characteristics are reported in Table 1. Participants could have multiple roles, for example, clinician and advocacy group member.

**Table 1:** Respite service providers and stakeholders characteristics.

*N* = 41	*n*	%
Age		
20–35	13	31.71
36–51	13	31.71
52+	15	36.59
Gender identity		
Female	35	85.37
Male	6	14.63
Role (could have more than 1 role)		
Service provider (e.g., respite worker, clinician, coordinator)	26	63.41
Stakeholder		
Policymaker or administrator	14	34.15
Community/advocacy group or family member	5	12.20
Time in role (years)		
Mean		7.78
Range		0.6–60
Province		
Manitoba	32	78.05
Ontario, Alberta or Saskatchewan	9	21.95

### 3.2 Themes

Themes are presented under two overarching sections: (A) challenges with the current respite care system; and (B) recommendations for an ideal respite care system. Subthemes within each theme are noted in **bold** font. Within the challenges and ideal respite sections, themes are ordered to reflect the flow of process steps from seeking respite care to outcomes of obtaining it or not. Findings also are visualized in the process maps (Figure 1A,B) providing a system view of themes (columns) shown as phases through the respite care process. Rows in the maps identify the different functional roles (e.g., families, funder, respite worker) within the system. Common process map shapes/symbols such as start and finish steps, activities, roadblocks and decisions represent subthemes or steps within each phase. Arrows and lines depict the direction and flow of the process, whereas solid lines depict smooth navigation, while dotted lines depict difficult navigation (see Figure 1 Key).

Figure 1(A) Respite care system cross‐functional process maps—current state themes. (B) Ideal state process map.

**Figure:**
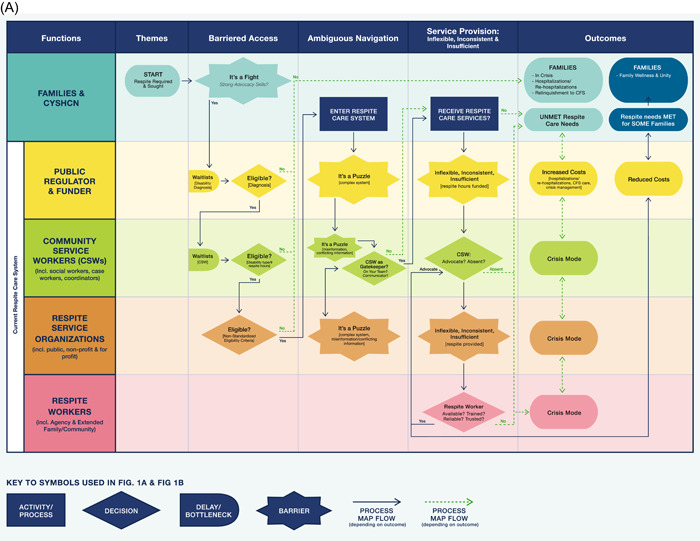

**Figure:**
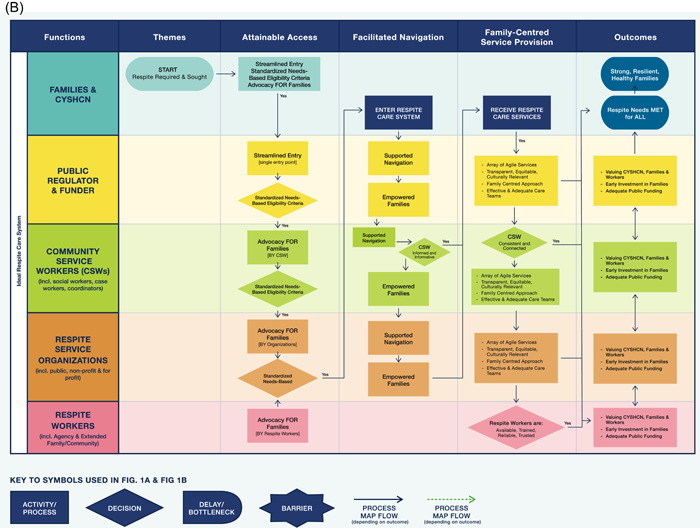

#### 3.2.1 Challenges within the current respite care system

Four key challenges themes emerged across multiple respite system functions: barriered access, ambiguous navigation, service provision: inflexible, inconsistent and insufficient and outcomes (respite needs unmet/met). Notably, public funding issues and the critical role of Community Service Workers (CSWs) were mentioned across three of these themes. Quotes within the text are examples of challenges shared by most participants. The term CSWs here and going forward includes coordinators, caseworkers and social workers.

Figure 1A current state process map highlights disconnects, where families of CYSHCN are shown functioning *outside* the respite system (see functions column), while funders, CSWs, respite service organizations and respite workers are shown as functioning within the system. The myriad of solid and dotted lines and arrows display key interactions between various functional roles and phases. The convoluted, chaotic family journey through the system in relation to activities that are performed in parallel by different functions^49^ results in uncertain outcomes of respite needs being met for some families, but unmet for others.

##### Barriered access

For families, the first step in accessing respite is to obtain a formal diagnosis for their CYSHCN from a medical professional. However, participants noted there were *‘**waitlists everywhere**’*, as some families wait months for a diagnosis and newcomer families wait for respite contract renewals. Backlogs in families being assigned a CSW further delay assessments for respite.

Access is further impeded due to a ‘who fits in the box’ approach to meet **eligibility criteria** for publicly funded respite. For instance, CYSHCN who do not ‘have the right disability’ such as a physical disability (e.g., cerebral palsy) versus an intellectual disability.

In most cases, I would say that the family feels like it's not easy to find and access respite, especially if they've fallen into one of these cracks where they are not eligible for public funding. [SP12]

Additionally, the nonstandardized **eligibility criteria** and funding entitlements across respite organizations, jurisdictions and regions further impacts access to respite.

If and when families receive a diagnosis and meet eligibility criteria, there is still a constant ‘***fight***’ to secure sufficient respite care. For families with strong advocacy skills and a supportive CSW, prospects of acquiring adequate respite care were greater. However, for families ‘who don't know how to be the squeaky wheel’, or fear advocating would impact other needs, their respite needs frequently go unmet.

##### Ambiguous navigation

Participants noted that **it's a puzzle** for families, SPs and organizations to navigate and operate within the respite system due to its ambiguity with misinformation/conflicting information and complexity.

It's just so complex …I've been doing it for a little while, but it is a mysterious web of systems and that is for me as a service provider and never mind being a lay person, never mind being a newcomer family, never mind having a language barrier and if you aren't attached to [organization], how are you supposed to know? (SP21)

CSW can, however, play a pivotal role in navigation, as in a sense **CSW are gatekeepers**. Whether families are able to effectively navigate through the system and receive adequate respite or not is significantly influenced by ‘who's on your team’ [SK10]. ‘But a lot of families didn't get that [extra respite] because their worker didn't tell them that they could. And you don't know what you don't know’ [SK12], highlighting communication gaps between CSWs and families.

##### Service provision: Inflexible, inconsistent and insufficient

SPs and SK spoke of the **inflexibility** in how funded respite hours are allowed to be used by families, stating, ‘there's no flexibility within the system. So, we're not even saying give me more hours, just let me manage the hours that I have better, so I don't burn out …’ [SK11].

Juxtaposed against inflexibilities, a strong subtheme depicted the current respite system as ‘*incredibly **inconsistent**’* across organizations and jurisdictions in the types and number of respite hours funded, and in how organizations interpreted respite service provision, resulting in unmet respite needs for families.

**Insufficiencies** in families receiving adequate support from their CSW and procuring respite workers often came up in the interviews. For example, having a CSW who was a strong **advocate** and in regular communication with families was more likely to lead to families’ respite needs being met. Whereas some CSWs were essentially **absent**, as families rarely heard from them or were unaware of who their CSW was. Insufficiencies in organizations due to limited provincial funding resulted in ‘this never‐ending battle of trying to find …keeping staff’ [SK03]. Moreover, low wages and limited training for **respite workers** led to high turnover which is further linked to the inability of families to trust their respite workers.

##### Outcomes: Respite care needs unmet/met

Given the inflexibilities, inconsistencies, and insufficiencies, the respite care system was described by numerous participants as operating in **crisis mode**, impacting both organizations, workers and families. For example, ‘huge’ caseloads for organizations and CSWs prevent them from providing meaningful care for families. Often, respite services are only funded and provided at ‘the last moment’ when families are ‘literally falling apart’. Not only is this approach detrimental to families, ‘it takes more time, more resources, more money, to provide services and then bring the client to the level that can function’ [SP22].

When families have **unmet respite care needs**, SP and SK spoke of ***families in a state of crisis***, leading to caregiver burnout, parental mental health issues, and limited capacity to care for their children as they would like to. With parents in crisis, siblings, sometimes referred to as ‘glass children, because the parents see right through them…’ [SP05] may be unintentionally overlooked by parents who are directing most of their time and energy to care for their CYSHCN. In severe cases, parental burnout and mental health issues led to emergency room visits and parents' **hospitalizations** or CYSHCN **rehospitalizations**. For example, ‘… we see kids frequently being readmitted, … strictly because parents just are burnt out. … We don't have any backup person for these parents, so they're parked in hospital’ [SP01]. When families felt they had no other recourse, **relinquishment to CFS** was used as a last resort.

There's been lots of situations where people have had to sign their children over to CFS. Because they don't know what else to do, and they're not being given the support that they need in their home to be able to keep their child at home. And like what an awful and horrible thing to have to do simply because you don't have the support that you need. [SK12]

Lastly, the consequence of families' unmet respite needs were said to result in **increased costs** for public funders, operating within a crisis model but yet not funding less costly respite care directly to families, as described by a SP.

I think it's absolutely ridiculous that, you know, where families with children who have severe disabilities that require 24/7, seven days a week care and the government or whoever is willing to pay thousands and thousands of dollars to have that child come into [CFS] care and be placed in a foster home and receive like high level funding, when they're not willing to put the services in that home so that that parent can stay home and take care of their child. [SP16]

In contrast, for **some** who had their **respite care needs met**, SP and SK identified this as supporting their **family wellness and unity** and reducing hospitalizations. Receipt of respite care was also noted as beneficial for CYSHCN, through relationship‐building opportunities and connections with others outside their nuclear families. Furthermore, with respite care needs met, parents were able to dedicate much‐needed time to their other children.

Participants also made numerous references to the advantages of adequately funding respite care for families in the early stages, resulting in **reduced costs** for public funders and better outcomes for families and CYSHCN.

If they [funder] provided the services in the early stage it will be easier, more beneficial for the family as well as for the system because you know like if they want to save money, they would be able to save money in earlier stage faster and better……than waiting until like… [A crisis]. [SP22]

#### 3.2.2 An ideal respite care system

Respite SP and SKs were asked to describe what an ideal respite system would look like for families of CYSHCN. The four main themes arising from this question provided solutions related to the current challenges identified above: Attainable Access, Facilitated Navigation, Family‐Centred Service Provision and Outcomes‐Respite for All. The call for more funding and the importance of cross‐system collaboration and communication were noted across several themes.

In contrast, to the current state process map (Figure 1A above), Figure 1B, the ideal state process map displays families functioning *within* the respite care system, alongside the other functional roles to depict a family‐centred system of respite care. Families' journeys through an ideal system would be smoother, more direct, with roadblocks and barrier activities being nonexistent. In an ideal respite care system, the ‘finish’ step or outcome of the process is that respite care needs are being met for all families of CYSHCN.

##### Attainable access

A strong theme across the interviews was that an ideal respite system would be one with ‘*more **streamlined***’ **entry** to respite care. ‘It would be a single point of entry, one door with families accessing information of the locations they live in, so families know more about the process…’. This would also allow families to only have to share their story once. Other participants suggested there should be **standardized eligibility criteria** by which families could reliably determine how much respite funding they would be eligible for, as currently, ‘…there's no rhyme or reason to it’ [SK12]. Others spoke of the necessity for **needs‐based** eligibility criteria, not solely based on having specific diagnoses, but on families' requirements.

Participants also recognized the role of **advocacy for families**, suggesting SPs and respite workers,

… need to start like stepping it up and start advocating more to say the government or like the higher ups about what is actually required, …what works best for the families that we work with… And now we need to start implementing what they need. And you know kind of start changing, changing the system. [SP16]

##### Facilitated navigation

According to SP and SK, navigating an ideal respite system would be a ‘straightforward’ process, with for example, a centralized database containing a list of vetted respite workers whom families could contact for provision of respite to their CYSHCN. The option of **supported navigation** services to guide families and respite staff would address current navigation challenges. This would include well‐**informed and informative CSWs** regarding available respite services and entitlements, actively sharing this information with families and ushering them through the system processes.

You really do, do need somebody and that's what their social worker [CSW] is. … So, they need to be aware of all that's out there and they need to be able to be **not** gatekeepers…. [SK03]

In turn, this would result in **empowered families** seamlessly navigating their way through the respite system:

I think that family members need to feel more empowered about identifying and navigating services that exist out there without going down a rabbit hole in Google to try and figure it out which is pretty much how our families find us. [SP17]

##### Family‐centred service provision

A family‐centred respite care system would offer an **array of agile services**, *a spectrum of options* that would be flexible, adjusting to the changing needs of CYSHCN and families, and *responding quickly … to keep people from going into crises* [SP14].

Specifically, addressing the challenges noted above with inconsistencies in current respite care entitlements, numerous SP and SK noted that an ideal respite care system would be **transparent and equitable**, meeting the needs of all CYSHCN and their families, including newcomer and Indigenous families, providing **culturally relevant** care.

Addressing the current challenges of CSW gatekeeping and lack of communication, one SK shared their knowledge of a transparent and equitable respite system in another country.

It was a very transparent system. You knew the more things that you had to deal with and the higher your score was, there was the likelihood of you getting that service…it was equitable. Everyone knew what they were entitled to. It didn't matter how good your worker was. [SK11]

A strong theme articulated by participants conveyed that an ideal system would include **effective and adequate respite care teams** made possible through adequately funding respite organizations/agencies that were delivering consistent care by ensuring respite staff were trained, well‐matched and connected with families. For example, teams in which families could rely on a **consistent** CSW who was available and well‐**connected** through *easier communication* to enhance family trust and relationship‐building. They would also include *a pool* of adequately paid, available, trained, reliable and trusted respite workers. This could be accomplished through *more generalized training, … that is more streamlined across agencies, not agency specific* and certified training to address the current crisis mode response of the respite system. Furthermore, allowance for and inclusion of extended family or community members within the pool of paid respite workers was recommended as an option for addressing respite worker supply and retention challenges.

Using a more comprehensive, **family‐centred approach**, would encompass focusing on what families need and allowing for an active role in decision‐making for their respite care plan through ‘…honouring the voice of the family. And then bringing in the voices of all those who support and coming up with a plan that, that everyone is on the same page about’ [SP10]. Furthermore, using this approach would better support families and avoid crisis situations ‘…to allow that family to stay together but function as a family’.

##### Outcomes: Respite for all

To ensure all families who require respite receive it, participants suggested a human‐rights framework from which an ideal respite care system could be approached, that of **valuing the CYSHCN, their family and those who provide respite care**, while using a family‐centred approach.

It's how you value people……that we're supporting right. If we value them, we pay the worker a living wage. We value them, we give the parents and the individuals some respite, if we value them. [SK11]

Participants felt strongly regarding the need for **early intervention and investment in families** in an ideal system of respite care, for example, building capacity and stability for families to care for their CYSHCN and preparing for the *marathon* of lifelong respite care and support. This approach would result in fewer undesirable outcomes such as family burnout, CFS placements and higher costs noted in the current respite system as shared by one participant.

I'm a hundred percent convinced that if families get what they need, when they need it, then you will save not only in care, like kids going into care. And being put in residential placements or whatever before families are ready, but health for everybody. All those people, not only the individual with the disability, but siblings, parents. Yes, health. So, they'll save money in the long run nationally. [SK03]

Early investment, however, requires **adequate public funding**. The call for *better funding* from public regulators and funders was a strong theme across the interviews for ideal respite care. At a system level, funding and providing adequate respite care preserves and maintains family functioning and well‐being, and if done earlier, can decrease costs for mental health and crisis‐based services, out‐of‐home care, and extended hospital stays. Similarly, for organizations providing respite services, *it's all about the funding* where adequate funding would augment resources to allow for adequate training, renumeration and supply of respite workers. This in turn would improve access to respite care for families of CYSHCN.

By valuing and investing in families with CYSHCN, through the provision of flexible, consistent, adequate and well‐funded family‐centred respite care, an ideal respite care system would demonstrate commitment to families, building **strong, resilient and healthy families**.

## 4 DISCUSSION

To the best of our knowledge, this study is the first to qualitatively explore the perspectives of respite SPs and SKs working with CYSHCN from multiple Canadian provinces. Two studies explored perspectives of Ontario respite SPs working with immigrant mothers,^22^ and families^50^ and a New Brunswick study included interviews with SKs working in the health, social and education sectors.^51^

### 4.1 Use of process mapping

While the intent of this study did not include a fulsome quality improvement process mapping approach,^52^ visual display of qualitative findings^53^ using cross‐functional or swim lane process maps^48^ provides new and novel insights (Figure 1A,B). Process maps have been used in quality of care research in oncology,^41^ and nephrology,^40^ however, to the best of our knowledge not previously been applied to respite care. Furthermore, incorporating outcomes (respite needs met/unmet) as the ‘finish’ step in the process map supports the project goal to inform respite care that is responsive and integrative, as well as a key message of the National Respite Care Project, ‘to understand respite as an outcome for the caregiver rather than simply as a service to the caregiver’, which is what would be required to truly provide respite to family caregivers.^54^ ^,p.1^

#### 4.1.1 Challenges within the current respite care system

The current challenges themes in this study align with much of the previously published international and Canadian qualitative literature on SP and SK perspectives regarding respite system challenges including access, navigation, service provision and unmet respite care needs.

The barriered access experienced by families as described by SK and SP including long waitlists,^8^, ^11^ narrowly‐defined eligibility^37^, ^51^ and having to ‘fight’ for support services,^22^, ^51^, ^55^ are unfortunately also common for families of CYSHCN in many jurisdictions. Hodgetts et al.^56^ ^,p.676^ suggest that assessments for respite eligibility are typically centred on existing services, ‘…which may not reflect the full spectrum of family needs’, leaving families and CYSHCN falling through the cracks^51^ and left without adequate respite care.

Navigation struggles have also been identified in other Canadian^22^, ^51^ and MB^8^, ^37^ studies with respite SP and families, along with knowledge exchange gaps between families with CYSHCN and respite providers,^22^, ^29^ thus we were not surprised by these findings. Unique to this study are the findings identifying the key roles of CSWs, case workers or social workers, who have the influence and authority to lead families into and through respite care systems, to ensure their respite care needs are met.

Similar to this study, concerns regarding respite service provision have been published previously in the international literature including service provision limitations and inefficiencies,^29^ inconsistent and inadequate respite worker training,^26^, ^34^ limited supply of adequately trained respite workers^37^ and funding inadequacies.^29^, ^51^ In MB, Manitoba Advocate for Children and Youth (MACY) previously identified the need for public investment and additional resources … ‘that realize the rights of children with disabilities in Manitoba and maximize value for money’.^8^ ^,p.10^ Furthermore, although many studies have identified service provision challenges within respite systems across Canada and beyond, participants in this study named current respite systems as operating in crisis mode, further supporting the need for change, and the aim of this study to inform responsive and integrative respite care for families of CYSHCN.

While SP and SK were not specifically asked about unmet and met respite care needs of families, questions similar to those asked in this study have been used previously to report on unmet caregiving needs in a Canadian context.^57^ Unmet respite care needs identified in this study frequently led to families being in crisis (Figure 1A), similar to other studies reporting caregiver burnout and elevated levels of psychological distress,^57^ with greater parental stress correlated with important unmet needs.^58^

Furthermore, acute‐care resource use,^59^ and hospitalizations or rehospitalizations of CYSHCN and/or parents, have previously been reported in the literature.^4^, ^5^

The need for some CYSHCN to go into foster (CFS) care to receive respite, while not as commonly reported, was also identified in the MACY report^8^ showing that over a 5‐year period, the child's disability was a contributing factor for 36% of MB children who entered into care. This is concerning, not only from a human rights perspective, which states children with a disability should not be separated from their families,^6^ but also given that children are most effectively cared for in‐home by their families.^8^

Ultimately, when respite care needs to go unmet, the outcomes can lead to significant healthcare costs, as highlighted by Cohen et al. using a population‐based approach for CYSHCN in Ontario.^59^

#### 4.1.2 An ideal respite care system

Inclusion in the present study of conceptualizations of an ideal respite care system with participants' solutions to address the current challenges identified above provides a blueprint for healthcare providers, policymakers and funders for moving forward towards respite systems change.

Although not as comprehensively as in this study, several qualitative studies report SP' and or SK' perspectives on both challenges with and recommendations for improved respite systems. Challenges included restrictive eligibility requirements,^51^ and funding gaps.^29^, ^51^ Recommendations encompassed single entry point access,^51^ navigation support,^22^, ^51^ communication and collaboration,^26^, ^51^ respite SP/worker training,^26^, ^29^, ^51^ person‐ and family‐centred care^26^, ^60^ and cultural responsiveness.^22^ However, only Khanlou et al.,^22^ Charlton et al.^51^ and King^50^ studies include perspectives of Canadian respite SP or SK.

Our findings for implementation of attainable access are supported by qualitative studies with SP/SK from the United Kingdom,^29^, ^34^, ^60^ Australia^26^ and New Zealand^55^ recommending single‐point‐of‐entry access,^29^, ^34^, ^51^ and accessible eligibility criteria.^26^ New findings in this study of the recommendation for respite access advocacy *for* families *by* respite‐providing organizations, CSWs and workers is warranted, as previous research has shown that carers, for example, parents, are least able to advocate for themselves with formal services when they are at a crisis point.^55^

While limited qualitative research exists on Canadian SP' and SK' recommendations for improving respite care, some of our findings align with calls for supported navigation services,^22^, ^50^ previously shown to be beneficial for CYSHCN.^61^ New findings by SK and SP in this study suggest an ideal respite care system would embrace family empowerment practices, which is supported by MB families who emphasized the need to recognize families as experts of their own needs.^37^ Also, previous qualitative studies with SPs/SKs from the United Kingdom,^29^, ^34^, ^60^ Australia^26^ and New Zealand^55^ support our findings recommending improved knowledge exchange with families and within teams.^26^

Notably, like our study, recommendations for improved service provision via more flexibility in amount and type of respite,^62^ using an array of service options for families were made more than 20 years ago by the National Respite Care Project, wherein participants noted that ‘typically, governments fund the individual plates on the buffet (i.e., single services) rather than the wide spectrum of choices that, in this case, is required. Governments should instead support the buffet approach – i.e., the infrastructure which enables the provision of a broad array of supports’.^54^ ^,p.6^

International qualitative studies with SP and/or SK^26^, ^29^, ^34^, ^55^, ^60^ also support our findings noting the need for transparency, culturally relevant care, and crisis prevention. Additionally, recommendations for transparent service information^56^ have been identified in Canadian studies.

Unsurprisingly, our findings calling for adequate and standardized training for respite workers,^34^ trusted respite providers,^55^ have been reported in qualitative studies with SP/SK internationally,^26^, ^29^, ^34^, ^55^, ^60^ including in Canada.^51^ New findings in this study suggest an ideal respite care system would support extended family/friends as paid respite workers. The use of informal support from extended family and friends is often sought out and helpful for families, although not always available, and becoming increasingly so as children age or conditions become more complex.^63^, ^64^ Recommendations by SP and SK in this study, and MB families^37^ to provide remuneration for informal respite carers such as family and community members could address insufficiencies with respite workers.

Again, similar to qualitative studies from the United Kingdom,^29^, ^34^, ^60^ Australia^26^ and New Zealand^55^ with SK/SP, the need for person‐family‐centred approaches^26^, ^34^, ^60^ support our findings for family‐centred respite that honours the voice of families.

When receipt of respite is the *outcome for* ^54^ family caregivers, they can rest and feel rejuvenated, and their respite care needs can be met. Hodgetts et al.^56^ ^,p.681^ suggest that ‘… needs are often well met *only if* families can actually learn about and access available services’, which supports the findings of this study, and the need to address the lack of accessible information for families. New findings in this study suggest an ideal respite care system would value CYSHCN, their families, and respite workers. Previous Canadian reports recommended viewing children with disabilities ‘…as equal members of the community…’,^8^ ^,p.9^ and valuing respite work as a career.^37^ However, the collective, more nuanced findings of this study reach beyond frequently reported logistical system improvement needs, highlighting the need for a more human rights‐based approach to respite care for families and CYSHCN.^6^, ^7^

Calls for adequate public funding for respite care from SP and SK in the United Kingdom,^29^, ^34^, ^60^ Australia^26^ and New Zealand^55^ support our findings for dedicated funding.^29^, ^34^ Public funders and regulators must act now to move to a new reality of respite care, using a responsive and integrative approach.

Furthermore, it is well established that receipt of respite care allows parents and families time for rest and self‐care and has been linked with improved marital quality,^65^ decreased stress in qualitative and quantitative studies involving MB siblings,^25^ parents^24^ and mothers^14^ of CYSHCN. Thus, in a responsive and integrative respite care system, accessible entry, followed by navigational support, and timely family‐centred provision of respite care makes for strong, resilient and healthy families (Figure 1B).

### 4.2 Limitations

There are several limitations to this study. We did not explore differences in respite care needs or service provision for different cultural groups. This study interviewed respite SPs and SKs. However, we did not interview SP and SK along with the families they serve to determine if families, SP and or SK share the same views in terms of best respite care for the CYSHCN. Also, longitudinal follow‐up with SP and or SK was not part of this study but would have been helpful to understand if and/or how respite service provision for CYSHCN has changed over time.

## 5 CONCLUSION

The evidence of unmet needs and the necessity for change within respite care systems in MB and across Canada has long been available, indeed well beyond the estimated latency of 17 years for new knowledge to reach routine clinical practice for example.^66^ The time for action to ensure a ‘respite is for everyone’ outcome is long overdue. The themes and process maps generated from our findings identifying respite system challenges and solutions can be used by funders and policymakers for planning and enhanced resource allocation, and by healthcare professionals, respite care providers and SKs to understand barriers and take action to improve respite care outcomes to meet the needs of all families and CYSHCN.

## AUTHOR CONTRIBUTIONS

Roberta L. Woodgate conceptualized the study. Roberta L. Woodgate and Sue Kirk contributed to the study design. Roberta L. Woodgate and Ardelle Kipling contributed to data collection. Roberta L. Woodgate, Ardelle Kipling and Corinne A. Isaak contributed to the data analysis and interpretation of the data. Roberta L. Woodgate and Corinne A. Isaak contributed to the drafting of the article. All authors critically revised it, gave approval of the final version to be published and agree to be accountable for all aspects of the work.

## CONFLICT OF INTEREST STATEMENT

The authors declare no conflict of interest.

## ETHICS STATEMENT

This study was approved by the University of Manitoba Health Research Ethics Board (#HS22685). All participants provided informed consent.

## Supporting information

Supporting information.

## Data Availability

Due to the nature of the research, supporting data are not available for ethical reasons. The participants of this study did not give written consent for their data to be shared publicly.
