# Supplementary material for: Challenges and recommendations for advancing respite care for families of children and youth with special health care needs: A qualitative exploration
Source: Health Expect. 2023 Sep 13;27(1):e13831. doi: 10.1111/hex.13831 (PMC10753137; doi:10.1111/hex.13831)
Supplement: Supplementary file 1 — Supporting information. [file HEX-27-e13831-s001.docx]

**Appendix**

Service Provider Interview Guide

**Introduction**

1. Could you please tell me a little bit about yourself and your/your organization’s role as a service provider for respite care?
2. Could you please give me a brief overview of the overall respite service environment in Winnipeg/Manitoba (or other jurisdiction for service providers outside of MB) for families of children requiring respite care?

**Service Providers’ Perceptions of Respite Care**

1. What do you think are the benefits of respite for children and their families?
2. How well do you think the current system of respite care is serving families of children requiring respite?
3. What challenges do you think families of children requiring respite face in accessing respite care services?
4. Can you share with me an experience of working with families who require respite services?
   - What have been some of the positive experiences? More challenging experiences?
5. What do you find that families want most with respite?
   - What are families using respite for?
   - What do you think families would most like to use their respite for?
6. What family members would benefit most from respite?

- What about the siblings of the child receiving respite? Would they benefit from respite? Please explain? Please tell me why?

1. Are there any groups of people that you think should be receiving respite but do not?
2. What do you think is unique about what you/your organization offer in terms of respite care, as compared to other respite providers?
3. What challenges do you as a service provider face in delivering respite care for families of children requiring respite?
   - What are some of the ways that you deal with those challenges?
   - What are some of the ways that your organization deals with those challenges?
4. During this time of the COVID-19 pandemic, how has delivering respite care changed for families of children requiring respite?
   - How have your interactions with families during these times changed?
   - How has the pandemic impacted the services and supports you are delivering?
   - Are families requesting anything different during these times? Please describe.
   - What are some of the challenges?
   - During this period, what has been helpful for you to carry out your job and what has not been helpful?
5. During this time of the COVID-19 pandemic, some families have asked if virtual respite could be arranged. What are your thoughts about virtual respite?

- What does virtual respite look like for you?
- What would be some of the challenges with virtual respite?
- What would you need to know in order to ensure that you carry out virtual respite properly?
- What guidelines would need to be in place to ensure that virtual respite is carried out properly?

**Recommendations for Responsive & Integrative Model of Respite Care**

1. From your perspective, what do you think is needed most to improve respite care services in Manitoba for families of children requiring respite?
2. Are there any changes that you would like to see that could help you do your job better?
3. Can you please describe what you think the ideal system of respite service would look like?
4. What criteria do you think would be important to consider in families seeking respite?
5. What do you think would need to happen that would make your ideal system of respite care possible?

**Ending Questions**

1. Can you please tell me what made you decide to take part in this study?

Is there anything else you would like to talk about that we did not talk about?

Are there any other questions we could ask service providers participating in this study that you think would be helpful to this study

How do you think we could get more people interested in this study?

Is there anything the research team could do/improve to make participating easier and more appealing to future participants?

Key Stakeholder Interview Guide

**Introduction**

- - **Ask demographic questions before this guide.**
  - **Obtain before interview via email:** Do you have any documents about how your program/model works in your organization that you could share? (It might help our interview process going forward).

1. Could you please tell me a little bit about yourself and your/your organization’s role related to respite care for families of children requiring respite care
   - Can you describe what some of these needs and conditions are that you mostly see in your program? (i.e. FASD, Autism, Cerebral Palsy)
2. Could you please give me a brief overview of the overall respite service environment in Winnipeg/Manitoba (or other jurisdiction for service providers outside of MB) for families of children with CCNC?

**Key Stakeholders’ Perceptions of Respite Care**

1. What do you think are the benefits of respite for children and their families?
2. How well do you think the current system of respite care is serving families of children requiring respite?
3. What challenges do you think families face in accessing respite care services?
4. Can you share with me an experience of working with families who require respite services?
   - What have been some of the positive experiences? More challenging experiences?
5. What do you find that families want most with respite?
   - What are families using respite for?
   - What do you think families would most like to use their respite for?

9. What family members would benefit most from respite?

- What about the siblings of the child receiving respite? Would they benefit from respite? Please explain? Please tell me why?

1. Are there any groups of people that you think should be receiving respite but do not?
   - Do immigrant and refugee populations have an impact on your respite services?
   - Can you speak to any special considerations for these populations with respect to respite?
2. What do you think is unique about what your/your organization’s role related to respite care, as compared to other organizations?
3. What challenges does your organization face in relation to your role related to respite care for families of children with CCNC?
   1. What are some of the ways that you deal with those challenges?
   2. What are some of the ways that your organization deals with those challenges?
4. During this time of the COVID-19 pandemic, how has providing respite care for families of children with CCNC changed?
   1. What impact has the pandemic have your role as a (Name of Role)?
   2. How has the pandemic impacted your policies and programs?
   3. How has the pandemic impacted the services and supports you are delivering?
   4. How have your interactions with families during these times changed?
   5. How have your interactions with service providers during these times changed?
   6. Are families requesting anything different during these times? Please describe.
   7. What are some of the challenges?
   8. During this period, what has been helpful for you to carry out your job and what has not been helpful?
5. During this time of the COVID-19 pandemic, some families have asked if virtual respite could be arranged. What are your thoughts about virtual respite?

- What does virtual respite look like for you?
- What would be some of the challenges with virtual respite?
- Do you have any concerns about virtual respite?
- What guidelines would you implement to ensure that virtual respite is carried out properly?

**Recommendations for Responsive & Integrative Model of Respite Care**

1. From your perspective, what do you think is needed most to improve respite care services for families of children requiring respite in Canada? What would be the steps involved if you could describe it?
   1. Is there anything unique about the context in which you work that would need to be accounted for in a new respite system?
2. Are there any changes that you would like to see that could help you do your job better?
   1. What would help you get more active in your role as an advocate for respite to change the system?
3. What criteria do you think would be important to consider in families seeking respite?
4. Can you please describe what you think the ideal system of respite service would look like?
5. What do you think would need to happen that would make your ideal system of respite care possible (at the individual level? At the systems level?)

**Ending Questions**

19. Can you please tell me what made you decide to take part in this study?

Is there anything else you would like to talk about that we did not talk about?

Are there any other questions we could ask key stakeholders participating in this study that you think would be helpful to this study?

Who else do you think we should talk to?

How do you think we could get more people interested in this study?

Is there anything the research team could do/improve to make participating easier and more appealing to future participants?
